# Role of Laboratory Tests in Enhancing Diagnosis and Treatment Planning for Dentists: A Review of Evidence-Based Practices

**DOI:** 10.7759/cureus.73671

**Published:** 2024-11-14

**Authors:** Hamad Albagieh, Mohammed N Aldrees, Abdulmajeed H Alshamrani, Salman M Alnadhari, Rashed M Almuwayni, Khalid F Alotaibi, Saud Alayed

**Affiliations:** 1 Dentistry, College of Dentistry, King Saud University, Riyadh, SAU; 2 College of Dentistry, King Saud University, Riyadh, SAU

**Keywords:** dentistry, diagnosis, laboratory tests, oral health, systemic conditions

## Abstract

The purpose of this review is to simplify and guide dentists through the main laboratory investigations frequently used in dentistry for diagnosis and patient management. It emphasizes the critical role of laboratory tests in dental practice, highlighting their significance for accurate diagnosis, effective treatment planning, and ongoing monitoring of oral health. Systemic conditions often manifest through oral symptoms, making laboratory assessments essential for identifying undiagnosed health issues that may impact dental treatment outcomes. A comprehensive literature search was conducted using PubMed and Google Scholar to gather relevant studies, ensuring a thorough examination of existing knowledge on the subject. This review defines commonly used laboratory tests, including complete blood count (CBC), coagulation tests, hemoglobin A1c (HbA1c), liver function tests, immunological assays, and microbiological and virological evaluations. Each test provides valuable insights into patient health, aiding in the detection of conditions such as anemia, diabetes, and liver disease, which can complicate dental procedures. By adopting an evidence-based, personalized approach to patient care, dental professionals can enhance safety and treatment success. This article underscores the necessity of integrating laboratory assessments into routine dental practice, promoting a holistic understanding of patient health and improving overall treatment outcomes.

## Introduction and background

In dentistry, laboratory tests are crucial for accurate diagnosis, effective treatment planning, and ongoing monitoring of a patient's oral health. These tests help dentists detect oral diseases, conditions, and abnormalities that may not be visible during routine clinical examinations, such as systemic conditions affecting oral health or underlying issues that could impact treatment outcomes. As was seen in previous studies, many patients may be unaware of their current medical conditions [1,2]. For instance, undiagnosed diabetes can lead to delayed healing after dental procedures [3], while untreated anemia can increase the risk of postoperative complications [4].

Another study has demonstrated that dentists and dental students possess a moderate level of knowledge about common diseases and their related laboratory investigations, However, these results also highlighted the need for additional courses and lectures to enhance awareness of the importance of lab investigations in dental practice. This includes a deeper understanding of the needs, interpretation, and procedures associated with these investigations, as well as which specific tests are mandatory for dentists to perform [5].

Furthermore, the implementation of laboratory tests promotes an evidence-based approach to dentistry, enabling clinicians to tailor treatment plans that consider each patient’s unique health profile. This individualized approach not only enhances patient safety but also improves the efficacy of treatments across a broad spectrum of procedures, from routine cleanings to complex surgical interventions. Understanding a patient's health status through laboratory evaluations can guide clinicians in making informed decisions about anesthesia use, medication prescriptions, and postoperative care [6].

The objective of this review is to offer a comprehensive overview of the commonly used laboratory tests in dental practice, understand their clinical importance, and clarify the relevance of these tests for dentists, which includes complete blood count (CBC), coagulation tests, hemoglobin A1c (HbA1c), liver enzymes and function, immunological tests, and microbiology and virology implementations.

## Review

Materials and methods

A comprehensive literature search was conducted using the National Library of Medicine’s Medline Database through PubMed in August 2023. The search employed the Mesh terms “Laboratory tests”, “Dentistry”, “Oral health”, and “Systemic conditions” to identify relevant articles published in English. Given the broad scope of the topic, all articles retrieved were considered for inclusion, regardless of study design or quality. The only exclusion criterion was if the article's content was not directly related to the role of laboratory tests in dental practice. Full-text versions of all relevant articles were obtained for review. Additionally, a thorough search performed on Google Scholar yielded fewer results. Lastly, reference lists from selected articles were examined for studies not initially captured in the database search.

Complete blood count (CBC)

It is one of the most common tests used in the medical field to evaluate a patient's overall health. A CBC includes a variety of readings, such as red blood cell (RBC) count, white blood cell (WBC) count, platelet count, hemoglobin level (Hb), mean corpuscular volume (MCV), mean corpuscular hemoglobin (MCH), mean corpuscular hemoglobin concentration (MCHC), red cell distribution width (RDW), and hematocrit. Additionally, it provides a differential count of WBCs, including neutrophils, basophils, eosinophils, monocytes, and lymphocytes. When reviewing a CBC most physicians typically rely on just four fundamental parameters, hemoglobin (Hb), hematocrit, WBC count, and platelet count (Table 1) [7,8]. A patient’s blood test results should always be interpreted using the reference values provided by the specific laboratory where the test was performed since reference ranges can vary between laboratories and are also influenced by factors such as gender and body mass index (BMI) [9].

**Table 1:** Normal value of CBC test in men and women. CBC: complete blood count, RBC: red blood cell, WBC: white blood cell. [8].

CBC	Normal value	
	Men	Women
RBC count (10^6 ^cell/mcL)	4.5-5.9	3.8-4.8
Hemoglobin (gm/dL)	14-17.5	12.3-15.3
Hematocrit (%)	41.5-50.4	35.9-44.6
WBC count (cell/mcL)	4,500-11,000	
Platelet (platelets/mcL)	150,000-450,000	

One of the most important parameters in CBC to diagnose anemia is the MCV which is an index used to measure the size of red blood cells. When the MCV is below 80 FL (femtoliter), red blood cells are categorized as microcytic, while values above 100 FL indicate macrocytosis. Normocytic red blood cells fall within the normal MCV range of 80-100 FL. Furthermore, the type of anemia can be classified accordingly with additional tests to discriminate between them as shown in (Table 2) [4,6].

**Table 2:** Types of anemia, etiology and tests to discriminate types of anemia. CBC: complete blood count, MCV: mean corpuscular volume, FL: femtoliter. [4,6].

Types of anemia	Etiology	Test to discriminate types of anemia
Microcytic anemia (MCV ˂80 FL)	Iron deficiency	Serum iron, ferritin
	Thalassemia	Hemoglobin electrophoresis
Normocytic anemia (MCV 80-100 FL)	G6PD (glucose-6-phosphate dehydrogenase)	Staining peripheral blood smears with methyl or crystals
	Sickle cell anemia	Sickledex
	Aplastic anemia	Erythropoietin levels
Macrocytic anemia (MCV >100 FL)	Folate deficiency	Serum folate levels
	Pernicious anemia	CBC serum vitamin B12

Importance in Dentistry

CBC provides valuable insights into the patient’s overall health status, aiding in the diagnosis of orofacial diseases. For example, it can identify systemic conditions such as anemia, infections, or blood disorders that may manifest with orofacial symptoms [4,10]. For patients with underlying medical conditions, a CBC can offer important information about their current health and potential risks during or after treatment such as postoperative infections and persistent bleeding [6]. Additionally, CBC is key in detecting anemia, which can impact the patient’s ability to undergo dental procedures. It also helps determine the severity of anemia, which is critical for tailoring appropriate treatment plans. Oral manifestations of anemia may include pale oral mucosa, atrophic tongue, glossodynia, petechiae, mucosal ulcers, gingival hypertrophy and bleeding (especially in aplastic anemia), dysphagia, taste changes, xerostomia, angular stomatitis, protrusive maxilla in thalassemia, and jaw radiolucency in sickle cell anemia. Pernicious anemia may also cause nerve paresthesia [4].

CBC plays a significant role in evaluating various conditions, including recurrent aphthous stomatitis; CBC alone cannot diagnose aphthous stomatitis, but it can help rule out underlying systemic issues or nutritional deficiencies that may contribute to recurrent ulcers [11]. CBC can also assist in excluding systemic causes of burning mouth syndrome, such as anemia or vitamin deficiencies [12]. For patients with persistent orofacial candidiasis, CBC can help assess if there are underlying hematological abnormalities or immune system issues contributing to the resistance of the infection [13].

Coagulation tests and their importance in dentistry

Coagulation tests are essential in dental practice to prevent excessive bleeding during and after surgical procedures. Understanding these tests is crucial for managing patients with bleeding disorders and those on anticoagulant therapy. Additionally, local hemostatic measures should be employed to manage bleeding effectively. By thoroughly evaluating coagulation status and implementing appropriate management strategies, dental professionals can enhance patient safety and minimize complications during surgical interventions [14].

Platelet Count

Platelets, which originate from proplatelets produced by megakaryocytes in the bone marrow, are small, nonnucleated cells. In healthy individuals, platelet counts typically fall between 150,000 and 450,000 per mm³, with an average lifespan of around 10 days in the bloodstream. Various regulators, such as nitrogen monoxide and prostacyclin, help maintain platelet function, and a healthy endothelium plays a crucial role in preserving its inactive state [15].

Reduction in platelet count or thrombocytopenia can lead to hemorrhages when platelet levels drop below 100,000 per mm³. Patients with counts below 50,000 demonstrate skin and mucosal purpura and bleed excessively with minor trauma. However, spontaneous bleeding is unlikely unless the platelet count falls below 20,000. Individuals with low platelet counts but normal platelet function typically do not experience spontaneous bleeding, though they may face prolonged bleeding after injuries, surgeries, dental extractions, or biopsies [16,17].

Prothrombin Time (PT) and International Normalized Ratio (INR)

PT evaluates the extrinsic pathway of coagulation, with normal values ranging from 11 to 13 seconds [5]. The INR is a standardized measure that helps assess bleeding risks. This is calculated by dividing the patient's prothrombin time by the laboratory's control value for prothrombin time [18]. In managing patients undergoing oral and maxillofacial surgery, monitoring the INR is essential for ensuring safe outcomes. By keeping the INR within a target range, healthcare providers can better manage and mitigate potential risks, thus enhancing patient safety and surgical success. An INR of 2.0 to 4.0 indicates a lower risk of bleeding during minor oral procedures, suggesting that simple extractions and minor oral procedures can typically be performed safely. while an INR exceeding 4.0 significantly elevates the risk of severe bleeding, necessitating the postponement of non-emergency surgeries until levels can be safely managed [19].

Activated Partial Thromboplastin Time (aPTT) and Thrombin Time (TT)

The aPTT test evaluates the intrinsic pathway of coagulation and typically ranges from 25 to 35 seconds in healthy individuals. Prolonged aPTT can suggest deficiencies in clotting factors, which increases the risk of bleeding during dental procedures. Thrombin time measures the time it takes to form a fibrin clot from fibrinogen, with normal values between nine to 13 seconds. An elevated TT may indicate abnormalities in fibrinogen or the presence of inhibitors affecting clot formation [17].

Bleeding Time (BT)

Normal BT ranges from one to six minutes [5]. Prolonged BT can indicate platelet dysfunction, necessitating caution during dental procedures. While BT is a useful screening tool, it is less reliable for predicting bleeding risk in deeper tissues compared to other coagulation tests [17].

Hemoglobin A1c (HbA1c)

Hemoglobin A1c (HbA1c) is a crucial blood test that reflects average blood glucose levels over the past two to three months, making it essential for diagnosing and monitoring type 2 diabetes. HbA1c is formed when hemoglobin in red blood cells binds with glucose in the bloodstream. Elevated HbA1c levels correlate with an increased risk of diabetes-related complications, underscoring its importance in assessing glycemic control and guiding diabetes management. Recent advancements have expanded its use not only for treatment adjustment but also for evaluating the quality of care and predicting the risk of diabetes complications. HbA1c levels are categorized as follows: a normal range is between 4% and 5.6%, levels between 5.7% and 6.4% indicate prediabetes, and levels of 6.5% or higher denote diabetes [20].

Importance in Dentistry

In dental practice, understanding a diabetic patient's HbA1c level is vital for assessing their overall health and potential risks associated with dental procedures. Elevated HbA1c levels can adversely affect the success of various dental treatments, from routine procedures like single crowns to more complex interventions such as implant placement. As HbA1c levels increase beyond the normal range (4-6.4%), the likelihood of successful oral procedures diminishes. The new Classification of Periodontal and Peri-implant Diseases and Conditions (2017) also emphasizes the significance of HbA1c levels in periodontal and peri-implant health, noting that elevated levels (≥7%) can influence the classification and management of these conditions [21].

The condition often leads to complications such as delayed wound healing, increased risk of infections, and a higher incidence of periodontal disease, necessitating careful management during dental procedures. Given the high prevalence of undiagnosed diabetes especially in regions like Saudi Arabia, where one in four adults is affected, dental professionals are in a unique position to screen for this chronic disease. Integrating diabetes screening into routine dental practice not only aids in early detection but also enhances the overall quality of patient care. By identifying patients at risk for diabetes, dental practitioners can tailor treatment plans, improve health outcomes, and potentially reduce the long-term complications associated with the disease. This proactive approach emphasizes the essential role of dentists in the broader healthcare system, highlighting the importance of interdisciplinary collaboration in managing chronic conditions like diabetes [3].

Liver enzymes and function

The liver plays a crucial role in metabolism, digestion, detoxification, and the removal of substances from the body. Common liver function tests include measurements of alanine aminotransferase (ALT), aspartate aminotransferase (AST), alkaline phosphatase (ALP), gamma-glutamyl transferase (GGT), serum bilirubin, total protein, and albumin. These tests can help identify areas of liver damage and based on the pattern of elevated results, aid in forming a differential diagnosis [8] (Table 3). Liver function test results should be consistent with the initial findings from a thorough patient history and physical examination. This review should explore relevant details, such as the patient's age, medical history, and any family history of inherited liver conditions. It’s also important to assess signs and symptoms of chronic liver disease, including jaundice, ascites, edema, encephalopathy, gynecomastia, pruritus, hepatosplenomegaly, and gastrointestinal bleeding [22].

**Table 3:** Liver enzymes, function, description and pathology in the liver. [9,23].

Indicators of liver function	Description	Pathology in liver disease
Bilirubin (Range: 2-17 µmol/L)	Breakdown product of hemoglobin -> unconjugated; Bilirubin (water insoluble) -> conjugated in the liver	Unconjugated (indirect): prehepatic (hemolysis); Conjugated (direct): liver disease
Albumin (Range: 35-50 g/L)	Protein produced by the liver	Reduced in liver disease and malnutrition
Liver enzymes	Description	Pathology in liver disease
ALT (alanine aminotransferase) (Range: 4-36 IU/L)	Found in the liver cells	Increased in hepatocellular damage
AST (aspartate aminotransferase) (Range 5-30 IU/L)	Found in liver, heart, and muscle	Increased in hepatocellular damage
Biliary enzymes	Description	Pathology in liver disease
ALP (alkaline phosphatase) (Range: 30-120 IU/L)	Found in bone and in the cells of bile ducts	Increased in cholestasis or biliary pathology
GGT (gamma-glutamyl transferase) (Range: 6-50 IU/L)	Found in many organs but highest in the liver (biliary)	Increased in cholestasis or biliary pathology, alcoholics

Bilirubin is a byproduct of hemoglobin breakdown, produced in the reticuloendothelial system. It is first released as unconjugated bilirubin, which then travels to the liver where an enzyme (UDP-glucuronyltransferase) converts it into its conjugated forms [23]. Albumin is produced by liver cells, and its production rate depends on the levels of colloidal osmotic pressure and the intake of dietary protein. In liver disease, serum albumin levels drop, indicating reduced production. If liver function is normal but albumin is low, it may be due to inadequate protein intake (malnutrition) or protein loss from conditions like nephrotic syndrome, malabsorption, or protein-losing enteropathy [9].

ALT is present in the liver in higher concentrations compared to other tissues like the kidneys, heart, and muscles. It is a cytoplasmic enzyme involved in catalyzing transamination reactions. Any form of liver cell damage can lead to a noticeable increase in ALT levels. AST is an enzyme that helps with transamination reactions and exists in two forms: mitochondrial and cytoplasmic, which are genetically different. It is most concentrated in the heart, but also found in the liver, skeletal muscles, and kidneys. ALP is found in the small intestine, kidneys, bones, liver, and placenta. It helps with lipid transport in the intestines and bone calcification. Most of the ALP activity in the blood comes from the liver, with about 50% coming from bones. GGT is a microsomal enzyme found in liver cells, bile ducts, kidney tubules, the pancreas, and the intestines. It helps transport peptides across cell membranes and plays a role in glutathione metabolism. While it is most concentrated in the kidneys, GGT activity in the blood mainly reflects the hepatobiliary system [23].

Importance in Dentistry

In general, patients with chronic liver disease have poor oral health and a variety of oral signs and symptoms. End-stage liver disease patients may experience excessive bleeding. Such individuals are more likely to experience postsurgical bleeding and oral symptoms such as gingival hemorrhage, petechiae, telangiectasia, and hematomas because they typically have decreased coagulation factors and thrombocytopenia [24,25]. It has also been noted that jaundiced mucosal tissue, malnutrition, and decreased gustatory function are common in cirrhosis patients [26]. Glossitis, loss of tongue papillae, and fissured tongue that was associated with xerostomia, along with angular cheilitis and candidiasis were the most encountered oral abnormalities in liver transplant candidates [27]. Liver diseases in early childhood, like biliary atresia, can lead to discoloration of permanent teeth. Increased bilirubin levels from cholestasis may cause the dentin layer of teeth to turn a greyish-green color. Additionally, developmental issues can result in enamel hypoplasia, showing up as white or yellowish spots on teeth [28]. An increased incidence of periodontal disease has been noted in patients with liver cirrhosis. The prevalence of periodontal disease ranged from 25% to 68.75% and apical periodontitis from 49% to 79% [29]. Sialadenosis, a condition characterized by painless, bilateral enlargement of the parotid glands, is commonly observed in patients with liver cirrhosis [30]. When diagnosing oral tumors, oral squamous cell carcinoma (OSCC) should be considered, as it is linked to alcoholic cirrhosis. Additionally, oral metastases from hepatocellular carcinoma should be a possibility, since patients with cirrhosis are at higher risk for this type of cancer. In patients with cirrhosis, oral manifestations often appear alongside other symptoms of liver dysfunction and may suggest that the condition is progressing or entering a decompensated stage. making them important indicators of worsening liver health. Monitoring these oral symptoms can help in the early detection of cirrhosis decompensation [31].

Immunological tests and their importance in dentistry

Autoimmune diseases are uncommon conditions caused by an abnormal immune response against the body’s own tissues and substances. These disorders are diverse, affecting multiple organs and cell types, with mechanisms that remain largely unknown [32]. While viral, bacterial, and microbial infections have been strongly linked to autoimmunity [33,34], several other factors such as genetics, age, gender, hormones, and reproductive status also influence their development [35,36]. Interestingly, smoking has been connected to autoimmune diseases, though snuff use has not [37].

Types of Immunological Tests

Direct immunofluorescence: This technique involves the application of fluorescent-labeled antibodies directly to tissue samples, allowing for the visualization of autoantibodies bound to specific antigens in oral tissues. Direct immunofluorescence is particularly useful in diagnosing conditions like pemphigus vulgaris, bullous pemphigoid, and mucous membrane pemphigoid, where autoimmune processes affect oral mucosa [38,39].

Indirect immunofluorescence: This method uses a two-step process where serum from the patient is tested against specific substrates that contain relevant antigens. Following this, a conjugated antihuman IgG is introduced to identify the autoantibodies from the patient's serum that have bound to the tissue substrate This technique is commonly employed in diagnosing various autoimmune conditions, enabling clinicians to visualize specific antibody-antigen interactions effectively [40]. This test offers valuable information regarding the presence of systemic diseases that can manifest orally.

Salivary biomarkers: Analyzing specific biomarkers in saliva, including cytokines and autoantibodies, can offer valuable insights into autoimmune processes and inflammatory responses occurring in the oral cavity. The use of salivary diagnostics is becoming increasingly popular due to their non-invasive nature, which allows for early detection of conditions such as Sjögren's syndrome. This approach enhances the potential for timely diagnosis and management of autoimmune diseases, making it a significant area of interest in clinical research [41].

Understanding the immunological profile of patients is crucial for dental professionals. Conditions such as Sjögren's syndrome can lead to significant oral health challenges, including increased risk of caries and periodontal disease. Early identification through immunological tests enables the implementation of preventive strategies and tailored treatments, ultimately improving patient outcomes. Incorporating these immunological tests into routine dental assessments allows for better management of autoimmune conditions, ensuring that dental care aligns with the patient's overall health status.

Microbiology implementations

Dental professionals frequently manage major diseases in clinical practice that are predominantly caused by microbial infections, such as dental caries, periodontitis, and odontogenic infections. However, unlike the approach taken in medicine for treating infectious diseases, the use of clinical microbiology laboratory testing in the diagnostic evaluation and treatment of dental patients currently appears to be limited oral microorganisms that can be collected from diseased areas or lesions within the oral cavity using saliva samples, dental plaque, mucosal smears, or soft tissue biopsies. Several commercially available methods exist for analyzing these oral microbial specimens, including microbial culture, nucleic acid hybridization and amplification techniques, and direct microscopic examination [42].

Recovering the microorganisms responsible for orofacial infections can be difficult due to the high risk of contamination by bacteria in saliva. Fast-growing bacteria like streptococci or staphylococci can interfere with the detection of slower-growing, clinically important species. The quality of the microbiological report depends heavily on how well the sample is collected. Because many bacteria involved in orofacial infections are sensitive to oxygen, it’s important to handle samples carefully to recover these anaerobes. To minimize contamination and protect these bacteria, pus should be collected via aspiration. If a swab must be used, it should be placed in a suitable transport medium. Since microbiological samples contain living bacteria, they should be sent to the lab quickly to preserve them. Once at the lab, a Gram stain can confirm whether the sample is pus, typically showing many white blood cells and a mix of both Gram-positive and Gram-negative bacteria [43]. Effective management of oral and systemic infections hinges on the careful collection, transportation, and testing of clinical specimens.

By utilizing various direct testing and culture methods, along with susceptibility testing, dental professionals and clinicians can accurately identify the causative pathogens and determine the appropriate therapeutic approach. Ensuring that specimens are handled correctly and that appropriate tests are applied is essential for achieving accurate diagnoses and improving patient outcomes. Table 4 and Table 5 summarize how these methods can be utilized in the management of oral infectious diseases [43,44].

**Table 4:** Specimen collection, transportation, direct testing methods for diagnosing various diseases and media used. TB: tuberculosis, AFB: acid-fast bacillus, ZN: Ziehl-Neelsen, DFA: direct fluorescent antibody, BAP: blood agar plates, CHO: Chinese hamster ovary, MAC: MacConkey medium, LJ: Löwenstein-Jensen, GC: Neisseria gonorrhoeae. [43,44].

Diseases	Collection	Transportation	Direct testing smears	Culture 2-5 days	Susceptibility (sensitivity)
Vesicles, Ulcer, Abscess, Wound, Mass, Thrush	Swab, Aspirate, Biopsy, Serology	Within 2 hours, Anaerobic transport media, Viral transport media, Tuberculosis (TB) in normal saline, Formalin for histopathology	Smear (Direct), Gram stain, AFB smear ZN stain, Giemsa stain, Silver stain, Fluorescent stains, Calcofluor white (Fungal), Auramine rhodamine (TB), DFA (Viral)	Culture, BAP, CHO, MAC, Anaerobic media (hemin, vitamin K), Liquid media, TB media LJ, GC media, Sabouraud dextrose agar	Disk diffusion, E-test, Automation system

**Table 5:** Bacterial clinical syndromes, their causative organisms, and the corresponding methods of diagnosis. TB: tuberculosis, AFB: acid-fast bacillus, GC: gonococcal. [43,44].

Bacterial clinical syndromes	Causative organism	Method of diagnosis
Scarlet Fever Erythrogenic toxins	Group A streptococci	Throat swab antistreptolysin O
Diphtheria	Corynebacterium diphtheriae	Throat swab toxin detection by Elek’s test
Vincent’s angina	Borrelia vincenti and Fusobacterium nucleatum	Throat swab by Gram stain
Actinomycosis	Actinomyces israelii branching beaded Gram-positive bacilli	Throat swab (solid and liquid media) 10 days incubation, sulphur granules, molar teeth colonies
Tuberculosis	Mycobacterium tuberculosis	Tissue, AFB smear and culture (normal saline) and Histopathology (formalin)
Syphilis	Treponema pallidum	Dark-field microscopy (primary) and serology, venereal disease research laboratory (VDLR), treponema pallidum hemagglutination (TPHA), fluorescent treponemal antibody absorption (FTA/ABS)
Gonococcal infection	Neisseria gonorrhoeae	Swab of the lesion, culture on GC media

Virology implementations

Viral diseases in the mouth affect oral tissues and can result from cellular damage or immune reactions to viral proteins. They often start suddenly and can cause blisters or ulcers. Some cases also show general symptoms like fever, tiredness, and swollen lymph nodes. While dental practitioners frequently encounter these conditions, diagnosing and managing them can be challenging. Some viral infections can lead to tumors, making early detection and referral for treatment important in dental care [45]. Oral lesions caused by viral infections are commonly seen by various healthcare professionals, including general practitioners, dentists, otolaryngologists, and dermatologists. These lesions can be difficult to diagnose due to their diverse appearances and the many potential viruses involved. Both Deoxyribonucleic Acid (DNA) viruses, like those from the Herpesviridae, Papillomaviridae, and Poxviridae families, and Ribonucleic Acid (RNA) viruses, such as enteroviruses and paramyxoviruses, can cause oral issues. Early detection and referral for treatment is crucial in dental care, especially for patients with human immunodeficiency virus (HIV) or acquired immunodeficiency syndrome (AIDS), as the presence of these lesions often indicates disease progression [46].

Several laboratory techniques are used to detect, isolate, and identify viruses in clinical samples, such as microscopy, culture, serology, and nucleic acid amplification. The choice of method depends on the suspected infection. Electron microscopy can give an initial identification by examining the size and shape of viral particles, but it requires further confirmation due to its low specificity. Viruses can be grown in cell cultures, for this, a lesion swab is placed in a viral transport medium that contains antibiotics to prevent contamination and maintain viral viability. The sample should reach the lab quickly, and processing starts after 24 hours to let the antibiotics work. The virus is then cultured in tissue cells, and its presence is indicated by cell changes that might take up to 10 days to appear, though this process can be faster with a high viral load. Alternatively, diagnosis can be confirmed by detecting a four-fold rise in antibody levels between acute and convalescent sera, but this method takes time and is less practical for immediate clinical use. Molecular techniques to detect viral DNA or RNA are becoming more common and are expected to improve the speed and accuracy of viral diagnosis [43].

Table 6 summarizes how these methods can be utilized in the management of oral viral diseases [43].

**Table 6:** Viral and fungal clinical syndromes, their causative organisms, and the corresponding methods of diagnosis. PCR: polymerase chain reaction, DFA: direct fluorescent antibody. [43].

Viral and fungal clinical syndromes	Causative organism	Method of diagnosis
Herpes labialis	Herpes simplex virus	Swab, viral transport media, cell line viral culture, DFA, polymerase chain reaction (PCR)
Chickenpox and shingles	Varicella zoster	Swab, viral transport media, cell line viral culture, DFA, PCR
Hand-foot and mouth disease and herpangina	Coxsackie virus A	Lesion swab, viral transport media, cell line viral culture
Measles Koplik’s spots mumps	Paramyxo virus	Throat Swab, viral transport media, cell line viral culture and serology
Infectious mononucleosis Burkitt’s lymphoma	Epstein-Barr virus	Monospot hetrophile antibodies, atypical lymphocytes
HIV Kaposi's sarcoma herpesvirus (KS HHV8)	Retrovirus	Serology, antigen detection, PCR
Oral candidiasis (thrush and Candida leukoplakia)	Candida albicans and others	Lesion swab for direct smear and culture on fungal media

## Conclusions

Laboratory tests play an essential role in dentistry by aiding in accurate diagnosis, treatment planning, and monitoring of a patient’s oral and systemic health. Important tests like complete blood count (CBC), coagulation tests, hemoglobin A1c (HbA1c), liver function tests, immunological assays, and microbiological and virological evaluations help identify underlying medical conditions that can impact dental procedures and patient outcomes. Integrating these laboratory tests into routine dental practice ensures a personalized, evidence-based approach, improving patient safety, treatment effectiveness, and overall health outcomes.
